# Predictive factors for intrauterine insemination outcomes: a review

**DOI:** 10.1186/s40738-020-00092-1

**Published:** 2020-12-11

**Authors:** Anabel Starosta, Catherine E. Gordon, Mark D. Hornstein

**Affiliations:** Department of Obstetrics and Gynecology, Brigham and Women’s Hospital, Harvard Medical School, Massachusetts Boston, USA

**Keywords:** intrauterine insemination, IUI, infertility, total motile count, sperm parameters, ovulation induction

## Abstract

**Purpose:**

Intrauterine insemination (IUI) is a frequently utilized method of assisted reproduction for patients with mild male factor infertility, anovulation, endometriosis, and unexplained infertility. The purpose of this review is to discuss factors that affect IUI outcomes, including infertility diagnosis, semen parameters, and stimulation regimens.

**Methods:**

We reviewed the published literature to evaluate how patient and cycle specific factors affect IUI outcomes, specifically clinical pregnancy rate, live birth rate, spontaneous abortion rate and multiple pregnancy rate.

**Results:**

Most data support IUI for men with a total motile count > 5 million and post-wash sperm count > 1 million. High sperm DNA fragmentation does not consistently affect pregnancy rates in IUI cycles. Advancing maternal and paternal age negatively impact pregnancy rates. Paternal obesity contributes to infertility while elevated maternal BMI increases medication requirements without impacting pregnancy outcomes. For ovulation induction, letrozole and clomiphene citrate result in similar pregnancy outcomes and are recommended over gonadotropins given increased risk for multiple pregnancies with gonadotropins. Letrozole is preferred for obese women with polycystic ovary syndrome. IUI is most effective for women with ovulatory dysfunction and unexplained infertility, and least effective for women with tubal factor and stage III-IV endometriosis. Outcomes are similar when IUI is performed with ovulation trigger or spontaneous ovulatory surge, and ovulation may be monitored by urine or serum. Most pregnancies occur within the first four IUI cycles, after which in vitro fertilization should be considered.

**Conclusions:**

Providers recommending IUI for treatment of infertility should take into account all of these factors when evaluating patients and making treatment recommendations.

## Introduction

Approximately 12–18% of couples in the United States struggle with infertility, with 20% of infertility caused solely by male factors and 30–40% of infertility caused by a combination of male and female factors [1, 2]. Intrauterine insemination (IUI) is a commonly used method of assisted reproduction for patients with mild male factor infertility, anovulation, endometriosis, and unexplained infertility [3]. In vitro fertilization (IVF) is generally used for severe male factor infertility [4].

Many factors affect IUI outcomes, including infertility diagnosis, semen parameters, and stimulation regimens. In this article, we review the current evidence regarding how patient and cycle specific factors affect IUI outcomes, specifically clinical pregnancy rate (CPR), live birth rate (LBR), spontaneous abortion (SAB) rate, ectopic rate, and multiple pregnancy rate. Couples may have multiple contributors to their fertility and IUI outcomes. Factors such as infertility diagnosis cannot be appropriately understood without considering other contributors such as semen parameters and stimulation regimen. Despite these limitations, we attempt to stratify the data by paternal, maternal and cycle factors in order to most rigorously draw conclusions. The current data on variables leading to IUI success are very heterogeneous. This review is meant to offer a thoughtful and concise interpretation of the data to help guide practice patterns and patient counseling. We aim to address each of the following questions:

1. How do paternal factors such as total motile count, inseminated sperm count, DNA fragmentation index, age and body mass index affect pregnancy outcomes?
2. How do maternal factors such as infertility diagnosis, body mass index and race/ethnicity affect pregnancy outcomes?
3. How do cycle factors such as stimulation regimens, ovulation trigger medication and timing, total number of cycles, and IUI procedure affect pregnancy outcomes?

## Methods

For this review, we first performed a computerized search of the published literature, limited to English language literature and conducted between April and November 2020. Databases searched were PubMed and Ovid. MeSH keywords used for the search included: “sperm count,” “ovulation induction,” “ovarian stimulation,” “body mass index,” “obesity,” “healthcare disparities,” “clomiphene citrate,” “letrozole,” “gonadotropins,” “polycystic ovary syndrome,” and “endometriosis.” Additional search terms included: intrauterine insemination, IUI, total motile count, post wash sperm count, stimulation regimen, ovulation trigger, cycles, paternal age, paternal body mass index (BMI), maternal BMI, and cervical factor. Literature searches identified retrospective and prospective cohort studies, randomized controlled trials, and systematic reviews and metanalyses. Upon reviewing the content of the retrieved articles, we also utilized their references to identify additional articles of interest. A total of 220 articles were reviewed, 102 of which were selected for inclusion. Article selection was initiated by AS (Anabel Starosta) with guidance and final approval by CG. We prioritized the most recent and relevant publications with inclusion of all randomized control trials, case control studies, retrospective and prospective cohort studies, and well-designed observational studies that contributed to the literature. Case reports, case series and meta-analyses were excluded.

## How do paternal factors such Total Motile Count, post-wash sperm count, DNA Fragmentation Index, age and BMI affect pregnancy outcomes?

Infertility results from male factor alone approximately 20% of the time [5] although the epidemiological evidence is limited [6]. IUI is an effective treatment for mild male factor infertility [3]. Even when other infertility etiologies are suspected, paternal factors such as total motile count (TMC), post-wash sperm count, and DNA Fragmentation Index (DFI) are important considerations in predicting IUI success rates. Paternal age and BMI may also impact clinical outcomes. However, the ability to predict pregnancy based on sperm parameters alone is limited, as outcomes also depend on female factors, such as age and infertility diagnosis, and stimulation regimens, which will be discussed in subsequent sections.

### Total Motile Count

There are conflicting recommendations in the literature regarding the threshold value for total motile count to best achieve pregnancy in IUI cycles. Many retrospective studies have evaluated cycle outcomes based on sperm parameters across a variety of ovulation induction (OI) regimens and infertility diagnoses. While this heterogeneity limits direct comparisons, most studies have demonstrated cut-offs for TMC of 5–10 million sperm. A 2001 study evaluated 1039 couples ≤43y who underwent 3,479 IUI cycles with natural cycles or ovulation induction with gonadotropins or clomiphene citrate (CC) to determine the prognostic factors for achieving pregnancy rate [3]. The authors found increased pregnancy rates at a threshold of 10 million. During the first IUI cycle, 1.5% of couples with TMC < 10 million conceived compared to 10.5% and 12.0% of patients with TMC 10–30 million and > 30 million, respectively. Studies investigating IUI with natural cycles or ovulation induction in couples with male factor infertility alone have also shown similar results. A randomized controlled trial (RCT) of 308 natural and ovulation induction IUI cycles showed a threshold effect at TMC of 10 million, with no improvement in pregnancy rates with ovarian stimulation among couples with TMC < 10 million [7] while two retrospective studies of 4,056 [8] and 2,062 [9] natural and OI/IUI cycles, respectively, showed a lower threshold of 5 million. Some authors have found lower cut-offs, with one study setting thresholds as low as 0.3 million [10] and another at 1 million [11], although these findings have not been widely replicated. A recent retrospective study of 310 women undergoing 655 IUI cycles found no live births in the 28 IUI cycles when the prewash TMC was < 2 million [12], although specific female infertility diagnoses in this group were not identified. These findings have led some authors to recommend proceeding with IVF at TMC less than 10 million [3, 7, 13] while others consider a lower threshold of 5 million [8, 14, 15].

### Post-wash sperm count

Post-wash total sperm count has been studied as a predictor of IUI success. A threshold value of 1 million has been observed in multiple studies [14, 16, 17]. However, a retrospective study of 3200 women undergoing 9963 cycles reported a higher threshold value, with decreased pregnancy rates when < 2 million sperm were inseminated [18]. The lowest post-wash sperm count which resulted in pregnancy was 0.8 million and pregnancy rates did not increase with higher sperm counts once the post-wash sperm count reached 4 million. A study of 1038 IUI cycles found increased pregnancy rates with a TMC threshold of 5 million and post-wash threshold of 1 million, with the highest pregnancy rates among women with ovulatory dysfunction and cervical factor infertility [14]. Semen preparation for insemination may be achieved with a number of methods. There is no data suggesting a difference in pregnancy or miscarriage rates across swim-up, wash and centrifugation, and density gradient preparation techniques [19]. The findings support the role of paternal factors in IUI outcomes while reinforcing the need to evaluate female factors in conjunction with sperm parameters.

### DNA Fragmentation Index

Altered sperm DNA structure may be a contributor to male infertility and is not characterized by typical sperm parameters, such as those discussed in the sections above. As a result, sperm DNA abnormalities can be expressed as the DFI with a DFI > 30% considered abnormal [20]. Increased DNA fragmentation has not been shown to correlate with conventional semen parameters when using Sperm Chromatin Dispersion (SCD) assays [20]. Furthermore, while high DFIs reflect sperm DNA alterations, clinical studies assessing the correlation of DFI determined by SCD with pregnancy rates in IUI cycles show conflicting data. A 2019 study of 1185 IUI cycles evaluating the impact of DFI on pregnancy outcomes found no difference in CPRs, although miscarriage rates were positively associated with higher DFI (27.3% high DFI; 14.6% middle DFI; 4.9% low DFI) [21]. A smaller study of 100 IUI cycles showed no difference in pregnancy rates despite a negative correlation with sperm motility [22]. Conversely, a study of 387 IUI cycles found lower biochemical pregnancy, clinical pregnancy, and delivery rates for couples with DFI > 30% compared to couples with DFI ≤ 30% [23]. Given that the most recent study in 2019, which also evaluated the largest number of cycles, showed no difference in pregnancy outcomes by sperm DFI levels, we do not routinely perform DFI testing for male partners undergoing IUI cycles.

### Paternal Age

The negative impact of advancing maternal age on fertility has been well documented. While advancing paternal age, especially > 40y, has been proposed to influence reproductive outcomes such as increased rates of preterm birth [24], spontaneous abortion [25], autism spectrum disorders [26], and infertility [27], the documented effects of paternal age are inconsistent across several cohort studies. A study of 2204 IUI cycles among women < 38y with BMI < 27 and no evidence of polycystic ovary syndrome (PCOS), tubal disease or endometriosis and men 25-56y (mean male age 34.3y) without severe male factor infertility found no impact of paternal age on pregnancy or miscarriage rates when stratified for maternal age and BMI, despite decreases in semen volume, concentration and motility with advancing paternal age [28]. Conversely, another study of 901 IUI cycles found decreased cumulative pregnancy rates in men > 35y compared to men < 30y after controlling for maternal age, ovulatory status, duration of infertility, and presence of asthenozoospermia and/or teratozoospermia [29]. The authors reported that in a multivariate analysis, male age ≥ 35y, duration of infertility > 3y and female ovulatory dysfunction were poor prognostic indicators.

### Paternal BMI

With rising obesity rates across the population, the impact of paternal BMI on fertility has been increasingly investigated. However, data on IUI outcomes by paternal BMI are limited. Multiple cohort studies have demonstrated that increasing BMI is negatively associated with semen parameters such as semen volume, concentration, TMC and morphology, with significant decreases observed in men with BMI ≥ 25 and the largest effects among men with BMI ≥ 30 [30–33]. One prospective study [34] evaluating the BMI of couples found increased risk of infertility when both the male and female partners were overweight or obese, with a direct relationship between increasing BMI and infertility. Rates of infertility were highest among couples who both had BMI ≥ 30, but couples had increased rates of infertility even when the female partner had a normal range BMI and the male partner was overweight or obese [34].

In summary, paternal and sperm parameter data support IUI for men with TMC > 5 million sperm and post-wash sperm count > 1 million. Higher post-wash sperm counts may increase pregnancy rates up to a threshold of 4 million. High sperm DFI reflects sperm DNA abnormalities but does not consistently impact pregnancy rates. Paternal age > 35y may negatively impact pregnancy outcomes but appears to have inconsistent effects. Paternal overweight status and obesity negatively correlate with fertility.

## How do maternal factors such as infertility diagnosis, BMI, and race/ethnicity affect pregnancy outcomes?

While patients seek infertility treatment for a number of causes, approximately 37% [35] of infertility results from solely female factors, and 30–40% of infertility is caused by a combination of male and female factors [1, 2, 35]. As such, pregnancy outcomes following IUI depend in part on maternal factors including age, BMI, race/ethnicity and infertility diagnosis. Based on maternal factors such as infertility diagnosis, pregnancy rates may be maximized with certain stimulation regimens or number of IUI cycles, which are further discussed in the subsequent section.

### Age

Advancing maternal age leads to decreased fertility due to diminished ovarian reserve and increased aneuploidy [36]. Studies evaluating the impact of maternal age on IUI outcomes after ovulation induction with gonadotropins or CC demonstrate diminished pregnancy rates in patients ≥ 40y, with rates of 4.1%-7% per cycle as compared to 13.7–17% in women < 40y [37, 38] across multiple infertility etiologies, including unexplained infertility, male factor and mild endometriosis. Stone et al. [18] evaluated 9963 IUI cycles among couples with predominantly unexplained infertility (50.3%), cervical factor, male factor and ovulatory factor infertility in which patients received either CC, gonadotropins, or a combination of the two for ovarian stimulation. Women < 40y had pregnancy rates 11.1%-18.9% per cycle while women 40-45y and > 45 had pregnancy rates of 4.7% and 0.5%, respectively [18]. Women < 30y required 1.99 cycles on average to conceive, while those > 40y required 2.24 cycles on average [18]. Although not all studies have found differences in CPRs when stratified by age, all have noted a trend of decreasing pregnancy rates with increasing age [14, 37, 39]. A randomized trial of couples with unexplained or male factor infertility compared pregnancy rates for natural and OI/IUI cycles and found age to be the only prognostic factor, with a 50% lower chance of conception for 38-year-old women compared to those who were 28y [39]. The Forty and Over Treatment Trial (FORT-T) investigated pregnancy rates and time to conception for ovulation induction IUI cycles with CC or Follicle Stimulating Hormone (FSH) versus IVF cycles in women aged 38-42y [40]. No differences in LBRs were observed between the CC/IUI and FSH/IUI groups. They noted significantly higher clinical pregnancy rates per cycle (24.7% versus 7.3%) and live birth rates per cycle (15.3% versus 5.1%) in the IVF group compared to those randomized to IUI [40]. As such, some providers recommend against IUI in this age group and discuss moving directly to IVF for women with unexplained infertility over the age of 38-40y.

### BMI

Obesity increases reproductive risks, including infertility due to menstrual dysfunction and oligo-anovulation [41–43]. Souter et al. [44] conducted a retrospective study examining the effect of BMI on 477 patients undergoing OI/IUI. Increasing BMI was associated with increased medication requirements (FSH) and fewer follicles produced per given FSH dose; however, there was no difference in mean number of cycles required to conceive, CPR, SAB rates, or multiple pregnancy rates [44]. Higher BMI was associated with increased endometrial thickness. The authors concluded that gonadotropin stimulation may overcome the ovulatory dysfunction that obese patients face when attempting to conceive naturally, enabling them to have success rates similar to patients of normal weight. Earlier studies have also shown no significant association between BMI and pregnancy rates [45, 46]. While increased endometrial thickness is associated with increased pregnancy rates in IVF, obesity leads to hormonal alterations within the endometrium, which can result in abnormal endometrial quality. As such, endometrial thickness such as that reported by Souter et al. may be a less reliable marker for pregnancy outcomes in obese patients [47]. Although obesity poses reproductive risks overall, the effects of obesity on IUI treatment outcomes do not reflect those obstacles.

Underweight status is also associated with increased rates of infertility due to hypothalamic amenorrhea and anovulation [48]. Patients who are underweight have an increased risk of small for gestational age babies, most notably for those who undergo ovulation induction [49]. Studies assessing the effects of underweight status on IUI outcomes are limited, but most providers recommend treating the underlying cause of underweight status prior to initiation of IUI or other fertility treatments.

### Race/Ethnicity

Disparities exist broadly within the healthcare system, with factors such as socioeconomic status, race and ethnicity affecting health outcomes [50, 51]. Black women have higher rates of infertility and longer duration of infertility prior to presenting to care compared to white women [52, 53]. Other minority groups such as Asian and Hispanic women also have longer times to infertility evaluation and may have different factors contributing to etiology of infertility compared to white counterparts [53–55]. Delays in diagnosis and treatment are significant given that longer duration of infertility is associated with worse pregnancy outcomes [37, 56]. Racial/ethnic disparities occur when using assisted reproductive technologies [57], but data on IUI outcomes are limited. The AMIGOS trial by Hansen et al. [56] evaluated OI/IUI outcomes in couples with unexplained infertility. An evaluation of the baseline characteristics of the study subjects found no differences in odds of conception or clinical pregnancy by race/ethnicity, but did find an association between Black race and lower odds of live birth. On the contrary, some retrospective studies have shown no differences in clinical pregnancy rates [58], multiple pregnancy rates or SAB rates [53] for Black, Hispanic and Asian patients compared to Non-Hispanic White patients, but one study found lower pregnancy rates for patients of American Indian decent [58]. Overall, evidence shows that race and ethnicity affect access to infertility treatments such as IUI and may affect outcomes, warranting further investigation.

### Infertility diagnosis

Several retrospective studies have examined the effects of infertility diagnosis on IUI outcomes. Sahakyan et al. [38] evaluated 613 gonadotropin OI/IUI cycles and after six cycles, found cumulative pregnancy rates of 84% for patients with ovulatory dysfunction, 57% for patients with unexplained infertility, 38% for patients with endometriosis, and 20% for patients with tubal factor infertility. Dickey et al. [59] evaluated 3,381 CC/IUI cycles and reported similar results, with cumulative pregnancy rates after four IUI cycles of 46% for ovulatory dysfunction, 38% for cervical factor/male factor/unexplained infertility, 34% for endometriosis, and 26% for tubal factor. After six cycles, CPRs increased to 65% for ovulatory dysfunction and 35% for endometriosis but remained unchanged for other diagnoses [59]. Results by Merviel et al. [14] demonstrated a similar trend with pregnancy rates per couple of 47.4% for ovulatory dysfunction and 55.6% for cervical factor infertility, but lower pregnancy rates for patients with endometriosis (10.7%) over varying numbers of cycles, ranging from 1 to 9. A randomized trial of IUI versus expectant management in patients with isolated cervical factor infertility demonstrated increased ongoing pregnancy rates with IUI (43% versus 27% ) and decreased miscarriage rates (7.7%; 19%) [60].

Overall, ovulatory dysfunction is associated with the highest cumulative pregnancy rates [14, 38, 59], suggesting that stimulation regimens successfully overcome ovulatory dysfunction. Although anovulation is associated with successful rates of conception after IUI, maternal factors affecting outcomes do not necessarily exist in isolation, and those with ovulatory dysfunction due to PCOS have an increased risk of obesity.

Tubal factor is associated with the lowest CPR after IUI, and Sahakyan et al. found that patients only conceived within the first two IUI cycles [38]. The authors suggest that these patients with tubal factor move onto IVF treatment after two unsuccessful cycles given these low pregnancy rates.

Although cervical factor infertility has been less studied than other infertility etiologies, IUI has been shown in an RCT to be an effective treatment for patients with isolated cervical factor infertility [60]. These findings, however, cannot be generalized to couples who may have multiple contributors to their infertility. Additionally, as shown by Merviel et al. [14], patients with both ovulatory dysfunction and cervical factor infertility are at increased risk for multiple pregnancies. Providers should consider reducing the risk of multiples in these patients through oral ovulation induction as opposed to OI with gonadotropins, as discussed below.

Endometriosis merits further discussion given the heterogeneity of clinical outcomes based on disease stage. For stage I-II endometriosis, cycle-specific pregnancy rates among women undergoing IUI ranged from 5.2%-6.5% compared to rates of 14% among women with unexplained infertility [37, 61]. Prado-Perez et al. [62] compared three groups of patients undergoing IUI: those without endometriosis, those with stage I and II endometriosis, and those with stage III-IV endometriosis. There was no difference in pregnancy rates by cycle between the first two groups (25.7% versus 22.7%), but there was a significant decrease in pregnancy rates in those with stage III-IV endometriosis (5.6%). Dmowski et al. [63] further investigated the role of IUI in this population, comparing pregnancy rates for couples with endometriosis treated with OI/IUI versus IVF (n = 313) and found higher cumulative pregnancy rates among those treated with IVF compared to IUI regardless of age or endometriosis stage, with the largest benefit to women older than 38y and in women with stage IV endometriosis. The IUI pregnancy rate per cycle was 11% with a cumulative pregnancy rate of 41% after six cycles, while the IVF pregnancy rate per cycle was 47% with cumulative pregnancy rate of 73% after three cycles, with no significant difference in rates of SAB or multiple gestation [63]. These results illustrate the significant impact of advanced endometriosis on pregnancy outcomes and suggest that providers may consider IVF over IUI for patients with advanced endometriosis.

In summary, maternal age and infertility diagnosis have a significant impact on IUI pregnancy outcomes, while maternal BMI may not have a large effect. Although the heterogeneity of studies limits the conclusions that may be drawn, the following can be considered. Given decreasing fertility with advancing age and lower pregnancy rates with IUI among older women, IVF can be considered for patients ≥ 38-40y. IUI is most effective for ovulatory dysfunction, unexplained infertility, and cervical factor infertility. Women with stage I-II endometriosis may benefit from IUI, while those with stage III-IV endometriosis and tubal factor have the lowest IUI pregnancy rates, and thus may benefit less from insemination. Increasing maternal BMI leads to higher medication requirements during OI/IUI but does not appear to impact pregnancy outcomes. Underweight women may benefit from increasing weight prior to initiating fertility treatments. Race and ethnicity affect access to infertility treatments and may negatively impact outcomes among minority groups.

## How do cycle factors affect pregnancy outcomes?

We will next examine how different cycle factors, specifically stimulation regimen, use of a trigger, number of cycles and insemination procedure affect pregnancy outcomes.

### Stimulation regimen

Accepted stimulation regimens to treat ovulatory dysfunction and infertility include CC, letrozole and gonadotropins. CC is a selective estrogen receptor modulator (SERM) which competes with estrogen for binding to the hypothalamic estrogen receptors, reducing negative feedback to the hypothalamus. This enhances hypothalamic Gonadotropin-Releasing Hormone (GnRH) secretion and subsequent gonadotropin release, driving ovarian stimulation [64, 65]. CC also leads to thickened cervical mucous and possibly altered endometrial quality [66]. Letrozole is an aromatase inhibitor which blocks estrogen synthesis, preventing the conversion of androstenedione and testosterone to estrone and estradiol, respectively [67]. This decreases negative feedback to the pituitary, increasing FSH release. Letrozole does not negatively impact the endometrium or cervical mucus production [68]. Gonadotropin therapies include human menopausal gonadotropin (hMG), which contains a combination of FSH and Luteinizing Hormone (LH), urinary-derived FSH (uFSH), and recombinant human FSH (rhFSH). Exogenous gonadotropins directly stimulate ovarian follicular development similar to the endogenous pathway. While debates occur over which ovarian stimulation regimen is optimal and one should consider individual patient factors, some conclusions can be drawn from the available evidence.

Several investigators have shown that pregnancy rates with FSH or hMG are superior to CC [69, 70] and letrozole in IUI cycles, although use of gonadotropins is associated with increased multiple pregnancy rates [71, 72] and risk of ovarian hyperstimulation syndrome (OHSS) [73]. Additionally, gonadotropins require frequent injections and are costlier compared to the oral regimens of CC and letrozole [70]. A retrospective study by Dickey et al. [70] compared CC alone, hMG alone, and sequential clomiphene followed by hMG and reported no difference in pregnancy rates per cycle between the CC-hMG group (22%) and hMG alone (18%), with significantly lower pregnancy rates in the CC group (11%). Given the comparable efficacy of combination CC and hMG with hMG alone, the authors concluded that the sequential regimen was superior, as it allowed for decreased utilization of hMG – reducing cost, number of injections, and monitoring. Diamond et al. [71] also demonstrated higher pregnancy rates and higher order multiple rates with gonadotropins over CC and letrozole in a randomized trial. They found no differences in miscarriage rates, ectopic rates, or OHSS. Given high rates of multiple pregnancies with gonadotropins, oral induction agents are typically preferred first line. Among patients with hypogonadotropic hypogonadism who lack endogenous gonadotropin formation, and thus would not respond to oral induction agents, the American Society for Reproductive Medicine (ASRM) recommends using gonadotropins for ovulation induction [74].

CC for ovulation induction with IUI is an established fertility treatment; however, the reported efficacy varies widely in the published literature. Pregnancy rates per cycle are reported to be 11.4%-21.5% [75–77] in ovulatory women and 9.7%-24.6% in anovulatory women [37, 78, 79]. Dovey et al. [80] conducted a retrospective cohort study with the largest series of CC data to date, totaling 4199 IUI cycles including patients who were both oligo-anovulatory and ovulatory. The authors stratified patients into age groups and found the following pregnancy rates for completed cycles: 11.5% for patients younger than 35y, 9.2% for patients 35 to 37y, 7.3% for patients 38 to 40y, 4.3% for patients 41 to 42y, and 1.0% for patients > 42y [80]. In the FORT-T study, pregnancy and live birth rates were equivalent among patients 38–42 years treated with CC or FSH and IUI, leading the authors to recommend the use of oral medications over injectables [40]. The Fast Track and Standard Treatment (FASTT) randomized trial [81] investigated time to conception and cost-effectiveness of IUI and IVF, comparing stepwise treatment of CC/IUI, FSH/IUI and IVF to accelerated treatment with IVF for couples who did not conceive after 3 IUI cycles. Pregnancy rates per cycle were 7.6% CC, 9.8% FSH and 30.7% IVF. Additional results showed shorter time to pregnancy (8 months accelerated versus 11 months conventional), fewer treatment cycles, and lower healthcare costs when patients proceeded from CC/IUI to IVF instead of first attempting FSH/IUI [81].

As letrozole has grown in popularity, multiple studies have compared its efficacy to that of CC. Diamond et al. evaluated 900 couples with unexplained infertility and randomized them to gonadotropins, CC or letrozole and IUI [71]. Results showed CPRs of 35.5% with gonadotropins, 28.3% with clomiphene and 22.4% with letrozole, and LBRs of 32.3%; 23.3% and 18.7%, respectively. CPRs and LBRs were lower in the letrozole group compared to gonadotropins alone or gonadotropins and CC. However, there were no significant differences between letrozole and CC alone. SAB rates, time to pregnancy, and rates of multiple gestation were also equivalent between letrozole and CC [71]. Letrozole does appear to be more effective than CC in patients with PCOS and those with at least Class I obesity. In a randomized trial of 750 women with PCOS treated with either letrozole or CC and regular intercourse, the letrozole group had higher CPRs (41.2% letrozole versus 27.4% CC) and cumulative LBR (27.5%; 19.5%) with no difference in multiple pregnancy or SAB rates [82]. LBR was higher for all patients receiving letrozole, with the largest difference between the letrozole and CC groups among patients with BMI 30.3–39.3 [82]. This data can be extrapolated to patients undergoing IUI cycles. One potential advantage of letrozole over CC was the proposed decrease in multiple pregnancies given primarily unifollicular development with letrozole [83]. However, the rate of multiples remains similar across studies as noted above. Early letrozole studies raised concern regarding its teratogenicity; however, a subsequent retrospective study demonstrated no difference in overall rates of congenital malformations [84].

### Ovulation medication and timing

In normo-ovulatory women, IUI may be performed either with a spontaneous LH surge or with a human Chorionic Gonadotropin (hCG) ovulation trigger. Spontaneous LH surge measured in the urine has been compared to administration of an hCG trigger in natural cycles in a retrospective study [85] and to CC-stimulated cycles in a randomized study [86]. Both studies found comparable pregnancy rates with urinary LH measurement versus use of an hCG trigger. The former study found no difference in live birth rates and the latter study found no differences in cumulative pregnancy rates over three cycles. A randomized study evaluated measurement of LH surge via serum blood draw, and demonstrated higher pregnancy rates in the spontaneous LH group as compared to use of an hCG trigger [87]. Cycle cancellation rates are higher when urinary LH is measured, possibly due to decreased sensitivity of urinary tests compared to serum tests, but cumulative pregnancy rates are unaffected. The available evidence suggests that LH monitoring is at least comparable to the use of an hCG trigger.

If an hCG trigger is used, most studies start ultrasound follicular monitoring by cycle day 11–13 and trigger ovulation when follicles reach ≥ 18 mm in mean diameter [88]. Most authors recommend avoiding an hCG trigger if a patient has four follicles ≥ 20 mm in mean diameter given the risk of OHSS, with consideration of cycle cancellation or conversion to IVF [86]. The hCG trigger may be given as a subcutaneous (SC) or intramuscular (IM) injection. IM and SC administrations result in similar serum hCG levels in women with BMI < 30 [89, 90]. Data from the IVF literature suggests that IM hCG injection is preferred over SC injection in obese women given significantly lower serum levels in the SC group [91]. Additionally, the typical needle length of 1.5 inches for IM injections may be insufficient to administer hCG in a number of these obese women, so tailored patient counseling is recommended.

### Cycle number

Most investigators recommend proceeding with IVF after three to four cycles given most pregnancies occur within the first four IUI cycles [37, 38, 80]. Studies have demonstrated up to 95% of pregnancies from OI/IUI with gonadotropins or CC are achieved within the first three cycles, with up to 98% [80] within the first four, resulting in little benefit derived from subsequent cycles. One study did show pregnancy rates up to 5.6% per cycle for cycles 7–9 with a cumulative pregnancy rate of 41% after the 9th cycle, but this was in a young patient population (< 35y) and only 12% of patients chose to continue with IUI after 6 total cycles [92]. The decision to continue IUI cycles should be guided by female age and ovulatory status, cost of treatment, and patient goals.

### Insemination procedure

Some practice variations in insemination procedure exist. In stimulated cycles, the timing of insemination varies from 24 to 40 hours after hCG injection with no difference in pregnancy rates [93–95]; as a result, exact timing may be guided by provider and patient preference. In natural cycles, IUI should be performed 24 hours after LH surge [96]. Single IUI is recommended. Double IUI, in which patients undergo insemination twice per cycle, has not been shown to increase pregnancy rates [97–100]. Following insemination, studies have evaluated whether 15 minutes of post-procedural rest is necessary. One randomized trial of 391 couples showed higher ongoing pregnancy rates (27% versus 18%) and live birth rates (27% versus 17%) among rested patients compared to mobile patients [101], while another randomized trial of 498 women reported no difference in cumulative pregnancy rates between groups [102]. Given the negligible risks and potential benefit, some providers may recommend post-procedural rest.

In summary, ovulation induction regimens include CC, letrozole and gonadotropins. Gonadotropins achieve the highest pregnancy rates, but also increase risk of multiple gestation and OHSS. CC and letrozole appear to have similar efficacy with no difference in CPRs, LBRs, SAB rates or multiple gestation among women with unexplained infertility. For patients with PCOS, letrozole increases LBR and CPR. For women who do not conceive on CC/IUI, proceeding to IVF rather than attempting FSH/IUI may be beneficial. For women ≥ 38y who undergo IUI, stimulation with CC or FSH achieves comparable outcomes. IUI may be performed with a spontaneous LH surge or with an hCG ovulation trigger with comparable pregnancy rates. If an hCG trigger is used, it may be given SC or IM, although IM is preferred for women with BMI > 30. Most pregnancies occur within the first three to four IUI cycles, after which alternate therapies should be considered. Insemination may occur 24–40 hours after hCG injection, or 24 hours after LH surge in natural cycles. Single IUI is preferred to double IUI. Brief rest after insemination may increase pregnancy rates.

## Conclusions

IUI is a frequently utilized and effective treatment for infertility, but outcomes depend on patient and cycle specific factors. Conclusions regarding outcomes and optimal management stratified by specific male, female and cycle factors are difficult to draw given the heterogeneity of studies and limited RCTs, but the following can be considered. Most data support IUI for men with a TMC > 5 million sperm, with some studies showing a threshold effect at a TMC of 10 million. Post-wash sperm count > 1 million is recommended, with increasing pregnancy rates as count increases but with a plateau in pregnancy rates after post-wash sperm count reaches 4 million. High sperm DFI reflects sperm DNA abnormalities but does not consistently impact pregnancy rates. Paternal overweight and obesity also contribute to infertility. Maternal obesity leads to increased medication requirements for ovulation induction, but does not affect pregnancy outcomes. Advancing maternal and paternal age negatively impact pregnancy rates, although the effects of paternal age are inconsistent in the literature while the negative impacts of maternal age are well documented. Given lower pregnancy rates with IUI, women ≥ 38y may benefit from IVF. While IUI data on race and ethnicity is sparse, there is evidence that disparities negatively impact access to fertility treatment and subsequent outcomes. Ovulation induction with gonadotropins, letrozole or CC may be used in conjunction with IUI to maximize pregnancy rates. Letrozole and CC result in similar pregnancy, SAB and multiple gestation rates and these treatments are recommended over gonadotropins given the high multiple gestation rate with the latter. For obese women with PCOS, letrozole is preferred over CC. IUI is most effective for women with ovulatory dysfunction and unexplained infertility, and least effective for women with tubal factor and stage III-IV endometriosis. Outcomes are similar when IUI is performed with an hCG trigger or a spontaneous LH surge, and LH may be monitored by urine or serum. When triggers are used, hCG may be given IM or SC, with IM injection preferred for obese women. Most pregnancies occur within the first four IUI cycles, after which IVF should be considered. Insemination may occur 24–40 hours after hCG injection, or 24 hours after LH surge in natural cycles. Single IUI is preferred to double IUI given similar pregnancy rates. Brief rest after insemination may increase pregnancy rates. Providers recommending IUI for treatment of infertility should take into account all of these factors. As inconsistencies and uncertainties in the IUI literature persist, this review raises questions that invite further research to maximize successful pregnancy outcomes.

## Data Availability

Not applicable.

## Abbreviations

ASRM: American Society for Reproductive Medicine
BMI: Body Mass Index
CC: Clomiphene Citrate
CPR: Clinical Pregnancy Rate
DFI: DNA Fragmentation Index
FASTT: Fast Track and Standard Treatment
FORT-T: Forty and Over Treatment Trial
FSH: Follicle Stimulating Hormone
GnRH: Gonadotropin-Releasing Hormone
hCG: Human Chorionic Gonadotropin
hMG: Human Menopausal Gonadotropin
IUI: Intrauterine Insemination
IVF: In Vitro Fertilization
LBR: Live Birth Rate
LH: Luteinizing Hormone
OHSS: Ovarian Hyperstimulation Syndrome
OI: Ovulation Induction
PCOS: Polycystic Ovary Syndrome
RCT: Randomized Controlled Trial
rhFSH: Recombinant FSH
SAB: Spontaneous Abortion
SCD: Sperm Chromatin Dispersion
SERM: Selective Estrogen Receptor Modulator
TMC: Total Motile Count
uFSH: Urinary-derived FSH
